# Differences of sucrose accumulation concentration and related genes expression between two sucrose accumulation types of *Actinidia eriantha*

**DOI:** 10.1038/s41598-020-77464-6

**Published:** 2020-11-24

**Authors:** Xiaobiao Xu, Guanglian Liao, Chunhui Huang, Min Zhong, Dongfeng Jia, Xueyan Qu, Qing Liu, Yanqun He, Yiqi Li

**Affiliations:** 1 College of Agronomy, Jiangxi Agricultural University/Kiwifruit Institute of Jiangxi Agricultural University, Nanchang, 330045 Jiangxi People’s Republic of China; 2 College of Forestry, Jiangxi Agricultural University/Jiangxi Provincial Key Laboratory of Silviculture, Nanchang, 330045 Jiangxi People’s Republic of China

**Keywords:** Plant breeding, Plant development, Plant physiology, Secondary metabolism

## Abstract

According to the investigation of wild *Actinidia eriantha* in Jiangxi province of China, we found that soluble solids content of fruit was lower than edible standard (14%). However, we found a high-sugar type *A. eriantha* line (code was ‘MM24’, test material) during investigative process at Nancheng county (E 116° 48′, N 26° 23′, 845 m). We sheared its scions to asexual reproduction in Fengxin County (rootstock was *A. deliciosa* ‘Miliang 1’ with 7 years old) and at the same time DUS (Distinctness, Uniformity and Stability) test was also carried out. There were uncontested differences between the two comparative genotypes according to the results of polyacrylamide gel electrophoresis, it can be judged as a new cultivar. In addition, there was great similarity on most important morphological and quality characteristics. While, there was difference on SSC, DM and TS between the two materials on ripen fruit, these indicators were much higher on test material than on control. The sugar degree assessment showed that the sugar degree of test material was strong and retention time was long. Further, no sucrose was found before DAF 135 d in test material and sucrose were significantly higher than in control only at DAF 165 d and DAF 175 d. The qRT-PCR results of sucrose-related genes showed that the relative expression levels of *AcSPS1*, *AcSPS3*, *AcSPS5* and *AcSUS5* genes were consistent with the sucrose accumulation trend, which was probably the main genes for the difference in sugar degree.

## Introduction

*Actinidia eriantha* is one kind of kiwifruit in which it belongs to Actinidiaceae family and includes 75 varieties and taxon^1^. Most of *A. eriantha* were found between 85° and 145°E and 0° ~ 50°N^2^. In last 20 years, kiwifruit has become one of the most favored fruit commercialized as it contains high ascorbic acid (AsA) content and other rich nutrients benefit to human being. *A. eriantha* is a unique germplasm resource in kiwifruit, not only the fruit has high nutrient, e.g., the content of AsA in the fruit of *A. eriantha* is 3 or 4 times higher than that of *A. chinensis*^3^. This novel berry fruit has very great development potential after currently most commercial planting of *A. chinensis* and *A. deliciosa*, the large abundant wild germplasm resources supply possible commercial cultivar selection for industry^4^. The fruit flavor is strong, the taste is mostly sour and slightly astringent^5^, high sugar degree is an important breeding target for *A. eriantha*, and it's very catered to Asians. Since 2009, germplasm resources investigation was conducted by the kiwifruit research team of Jiangxi Agricultural University, we collected more than 700 *A. eriantha* germplasm from Jiangxi Province of China^6,7^. We found a high-sugar type *A. eriantha* line in Nancheng county, Jiangxi province and its soluble solids content (SSC) of ripen fruit more than 19%, which was significantly higher than edible standard. It had a stronger resistance to adverse circumstance and better organoleptic quality compared to fruit of wild kiwifruit (*A. eriantha*). We sheared its scions to asexual reproduction in Fengxin County of China (rootstock was *A. deliciosa* ‘Miliang 1’ with 7 years old) and the DUS test was also carried out.

The high-sugar type *A. eriantha* cultivar is a very valuable material. It would contribute to our understanding of the regulatory mechanism of sugar accumulation in *A. eriantha* and more generally in kiwifruit. In this paper we introduced the main agronomic traits of fruit on high-sugar type *A. eriantha* line and determined the related genes expression, finally, discussed the main reason on sugar types during fruit growth, it would lay a theoretical foundation for studying the regulatory mechanism of sugar accumulation.

## Materials and methods

### Plant materials

**Figure 1:**
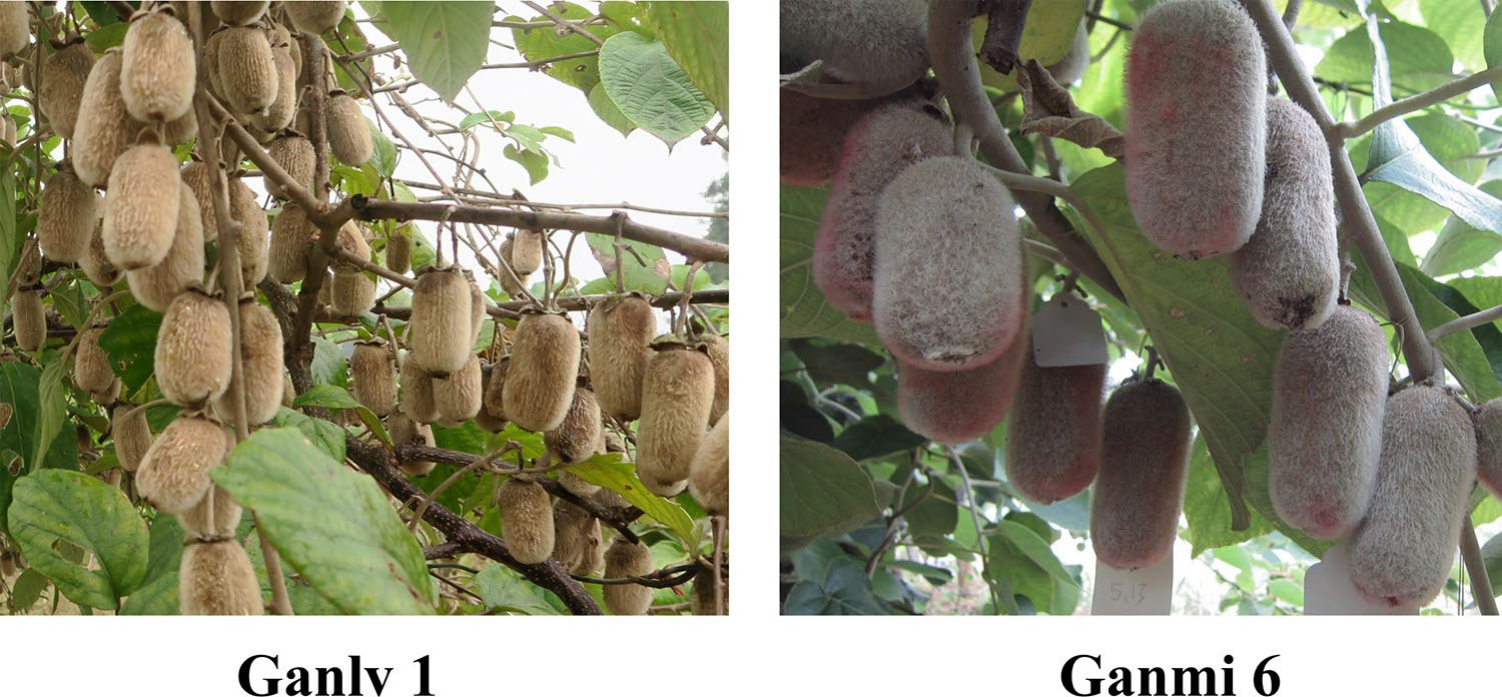
Fruit of test materials. They were cultivated in a kiwifruit orchard located at Fengxin County, Jiangxi Province.

Both ‘Ganmi 6’ (low-sugar type cultivar) and ‘Ganlv 1’ (high-sugar type cultivar) (Fig. 1) vines were cultivated in a kiwifruit orchard located at Fengxin County, Jiangxi Province. Both of them selected from wild *A. eriantha* on Nancheng county and they have similar genetic background. In this study, we sheared scions of two materials to asexual reproduction in Fengxin County (rootstock was *A. deliciosa* ‘Miliang 1’ with 7 years old). Ten sampling stages determined by the days after full bloom (DAF) were as follows: 25, 50, 75, 100, 125, 135, 145, 155, 165 and 175 DAF (harvesting time, soluble solid content (SSC) reached 6.5%^8^). We collected 50 fruit from both materials on the same dates, 10 fruit were used to measure the physiological parameters. All fruit samples were taken from kiwifruit germplasm garden of Fengxin county, Jiangxi province, China. The flesh of the fresh samples was dissected using a blade, frozen immediately in liquid nitrogen, and then stored at − 80 °C. In addition, 30 fruit were collected when it harvested, quality indexes of ripen fruit were determined two weeks after storage at room temperature.

### Phenological and morphological characteristics observation

It is useful for production by observe phenological of kiwifruit. The phenological of mutant was tested according to the methods of Xia et al.^9^. The time of begin flowering is defined that about 20% buds to open, the full flowering time is over 50% buds to open, time of blossom fall is that over 80% flower fall. Physiological maturity stage of fruit is defined that the SSC reached 6.5%. Twenty vines per genotype were examined and the main characteristics of leaves (shape, apex et al.), flowers (color, size arrangement of petals et al.) and fruit (size, shape et al.) following former report by Liao et al.^10^.

### Fruit quality evaluation and sugar degree assessment

The weight of fruit was tested immediately after harvest. After 2 weeks of storage at room temperature when the fruit had become edible^8^, Fruit quality evaluation included hue angle of flesh color (color-difference meter model is CHROMAMETERCR-400, the mean was taken from two measurements on opposite sides around the equator of each fruit immediately after careful skin removal), fruit dry matter (with slice thickness of 1 mm), soluble solids content (with numerically explicit sugar meter). In addition, the content of soluble sugar content (TS), titratable acid (TA) and AsA were measured by grinding the fruit pulp treated with liquid nitrogen^11,12^. At the same time, the sugar and acid compositions were tested with High Performance Liquid Chromatography with reference to the method of peach^13^.

Time-intensity (Osme) method was used for sugar degree assessment^14^. Each treatment was weighed 100 g ripe fruit for the sugar degree analysis. Twenty experienced sensory evaluation specialists taste the fruit and exchanged their observations in term of retention time and intensity. The assessment was replicated three times. The sugar degree intensity was divided as weak, medium and strong.

### Molecular genetic analysis

Molecular genetic analysis for *Actinidia* genotypes was based on microsatellite DNA molecular markers using PCR^8^. For the DNA genetic analysis, repeatable samples of the three compared kiwifruit materials were used. The process for DNA analysis included leaf sampling (0.1 g) per genotype and DNA extraction process via a modified CTAB method. The extracted DNA was quantified on agarose gel by comparison with report samples (DNA markers). The DNA quality and quantity were tested for appropriate molecular genetic analysis. For genome screening, six primer pairs were used to amplify dinucleotide tandems AG/CT and AC/GT. The primers pairs were developed in multilateral INCO-DC project and are appropriate, accurate and reliable for *Actinidia* molecular genetic analysis. PCR products were separated in an 8% polyacrylamide gel, 1.5 mm thick, and band visualization was possible by the silver nitrate method.

### Ploidy level analysis

The young leaves of about 0.5 cm^2^ sample were put on the slide, add 400 μl extract liquor to it and use a sharp blade to cross cut the sample, soak the sample for 3 min, then filter the sample into the sample tube with the filter net, and add 1600 μl DAPI dye, wait for 3 min, we can use the CyFlow Cube flow cytometer to detect the ploidy. With the known multiplier ‘Hongyang’ (2 ×), ‘Jinyan’ (4 ×), ‘Jinkui’ (6 ×) as the standard sample, set the voltage and flow rate before test the ploidy of sample^10^.

### RNA extraction and qRT‑PCR

Total RNA was extracted from kiwifruit flesh according to the method described by Wu^15^. RNA samples were subjected to analysis of RNA degradation and contamination using agarose gel electrophoresis; RNA purity testing using the OD260/OD280 ratio as measured standard using the NanoDrop system (IMPLEN, CA, USA). The cDNA synthesis kit (Thermo Fisher Scientific, MA, USA) was used, according to the manufacturer's instructions, to convert the total RNA samples into single-stranded cDNA, which were utilized for the Real-time qPCR analysis.

Based on previous experiments, we have identified members of the *AcSP*S and *AcSUS* gene family of kiwifruit (unpublished). The Real-time qPCR primers were designed by Primer 5 (Table 1). The PCR mixture contained 2.5 μL double distilled water (ddH_2_O), 5 μL SYBR Green I master mix (Asbio Technology, Inc.), 0.5 μL of the forward and reverse primers, and 1.5 μL cDNA template. The LightCycler 480 real-time PCR system with a 96-well plate was running to conduct the reaction. The conditions for the PCR amplifications were as follows: 95 °C for 5 min, followed by 45 cycles of 10 s at 95 °C, 20 s at 60 °C, and 20 s at 72 °C. The β-actin in the kiwifruit was considered as the control gene for normalization^16^. All analyses were repeated three times using biological replicates. The relative expressions were calculated using the 2^−ΔΔCt^ method^17^.

**Table 1:** Designed primers of quantitative real-time PCR.

Gene name	Forward primer sequence	Reverse primer sequence	Product size/bp	Annealing temperature/°C
*AcSPS1*	CTATCAATGACAAGAAGGGCGAAAA	GCAACGGTGAGCCTGAATCCT	158	63.6
*AcSPS2*	ATGCTTTTCACTGGTCACTCACT	CATCATACAGACGCCATTGCTC	199	58.2
*AcSPS3*	TTCTGAAGTTGGTCCTTTTGGG	TCGTCTTCAGCAACTTGTCCTC	165	61.2
*AcSPS4*	ACGAGCACCAAGCAGGAGATT	CCACAACATTGCTGAAGTCCATA	169	61.3
*AcSPS5*	GGAGAAGGAAAAGGGTGATGC	CCTGACCTCCAGTGTCTGAATC	191	58.4
*AcSUS1*	AACTTGGGATTACTCTGGGAACT	ATTTCTTGGTATGTGCTGGTGAT	175	58.5
*AcSUS2*	GGTGGCTTACAGAAGGCTCAG	CCACTGCCTAAACCTTTGCTC	195	59.2
*AcSUS3*	GTTGCTTTGGCAGTGAGGAGG	GGTTAGGCGACGACTTGAGAAA	198	61.5
*AcSUS4*	TCAAAGAATACAACTTGGATGGC	CAAGTGGCAAATGTTGGAAGC	187	59.4
*AcSUS5*	TTGGGCTATCCTGACACTGGT	CAATGAGAATGCGTGGAATGA	124	59.4
*AcSUS6*	ACACTGGTGGTCAGGTGGTTT	GCGAATGTTCTGCTCCGTAAA	189	59.8
*AcSUS7*	ATCAAAGCCCATTATCTTCTCCA	TGACTTCTTCACATCATTGTAACCC	145	60.1

### Date analysis

Statistical analysis for physical and quality characteristics was performed with analysis of variance over the materials and period with the SPSS statistical package (SPSS 17.0). Significance levels are determined per parameter.

## Results

### Biological characteristics and sugar degree assessment

**Figure 2:**
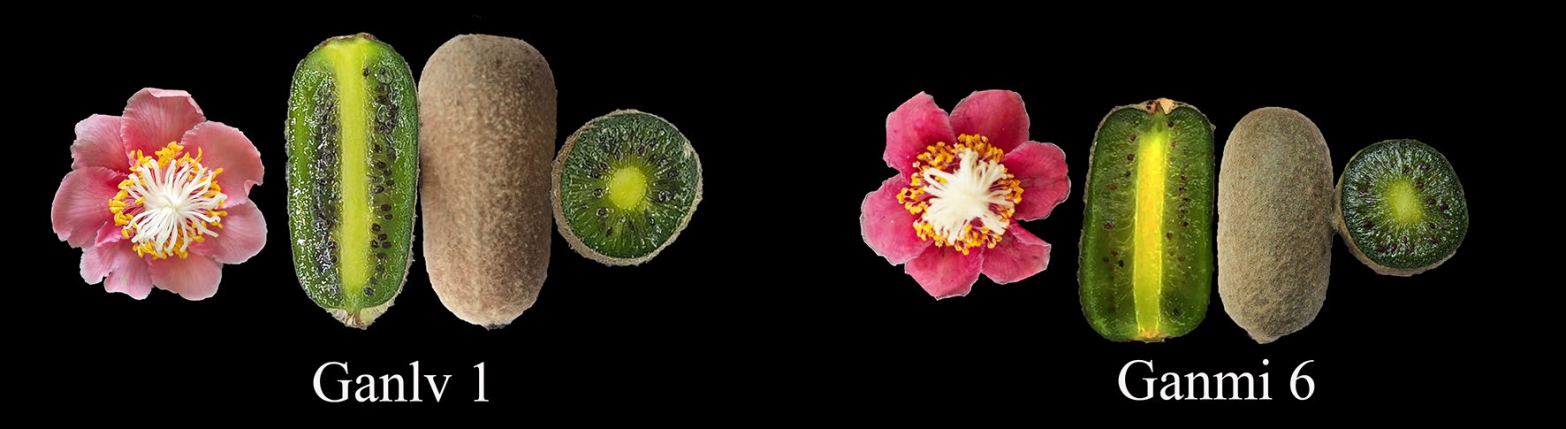
Morphology of flower, leaf and fruit of two materials.

We found that there is a similarity at the time of three flowering period (initial bloom stage, full bloom stage and fall bloom stage) and fruit matured period between ‘Ganmi 6’ and ‘Ganlv 1’ (Table 2). The comparative description of morphological characteristics between two materials for distinctness also presented in Table 2. We found that some characters of leaf and flower of ‘Ganlv 1’ were consist with ‘Ganmi 6’, such as shape of leaf, flower size, arrangement of petals and so on. There was difference on AsA, SSC, DM and TS between the two materials. Peals color and fruit shape of ‘Ganlv 1’ was different with ‘Ganmi 6’ (Fig. 2). Fruit mass from ‘Ganmi 6’ was larger (by 20.45% mean value) than ‘Ganlv 1’ in harvests. No difference was found between 3 years on characters of two materials. The sugar degree assessment showed that the sugar degree of ‘Ganlv 1’ was strong and sugar degree of ‘Ganmi 6’ was medium.

**Table 2:** Comparison of economic characters between the two materials.

Years	2016		2017		2018	
Cultivar	‘Ganlv 1’	‘Ganmi 6’	‘Ganlv 1’	‘Ganmi 6’	‘Ganlv 1’	‘Ganmi 6’
Initial bloom stage (Month. Day)	5.6–8	5.9–10	5.5–6	5.7–8	5.5–7	5.6–7
Full bloom stage (Month. Day)	5.9–13	5.11–14	5.7–10	5.9–11	5.8–11	5.8–11
Fall bloom stage (Month. Day)	5.14–15	5.15–16	5.10–11	5.12–13	5.12–13	5.12–13
Time of harvest (Month. Day)	10.24–26	10.27–28	10.24–25	10.26–28	10.22–25	10.23–25
Leaf: shape	Ovoid	Ovoid	Ovoid	Ovoid	Ovoid	Ovoid
Leaf: shape of apex	Acuminate	Acuminate	Acuminate	Acuminate	Acuminate	Acuminate
Leaf: color	Emerald	Dark green	Emerald	Dark green	Emerald	Dark green
Leaf: length/cm	16.78 a	17.18 a	16.91 a	17.23 a	16.90 a	17.31 a
Leaf: width/cm	11.56 b	12.07 b	11.86 b	12.09 b	11.88 b	12.11 b
Leaf: petiole length/cm	2.13 b	4.80 a	2.43 b	4.85 a	2.35 b	4.87 a
Flower: diameter	Small	Small	Small	Small	Small	Small
Flower: size	Small	Small	Small	Small	Small	Small
Flower: arrangement of petals	Overlapping	Overlapping	Overlapping	Overlapping	Overlapping	Overlapping
Flower: color of petals	Pink	Bright red	Pink	Bright red	Pink	Bright red
Fruit: average weight (g)	46.51 b	52.13 a	46.16 b	55.6 a	45.98 b	54.4 a
Fruit: shape	Ellipsoid	Oblong	Ellipsoid	Oblong	Ellipsoid	Oblong
Fruit: Fruit shape index	Micro convex	Micro convex	Micro convex	Micro convex	Micro convex	Micro convex
Fruit: length (cm)	6.28 a	5.26 b	6.35 a	5.36 b	6.33 a	5.39 b
Fruit: large width (cm)	2.58 b	2.98 a	2.42 b	2.91 a	2.52 b	2.93 a
Fruit: small width (cm)	2.48 b	2.92 a	2.12 b	2.89 a	2.23 b	2.91 a
Ripe fruit: SSC (%)	19.10 a	14.6 b	18.89 a	14.5 b	19.12 a	14.7 b
Ripe fruit: DM (%)	21.72 a	17.4 b	21.76 a	17.0 b	21.75 a	17.9 b
Ripe fruit: TA (%)	0.94 a	0.9 a	0.92 a	0.9 a	0.93 a	0.9 a
Ripe fruit: TS (%)	9.14 a	8.3 b	9.43 a	8.2 b	9.19 a	8.3 b
Ripe fruit: Hue (°)	132.4 a	124.3 a	135.26 a	124.3 a	139.25 a	124.7 a
Ripe fruit: AsA (mg/100 g)	662.1 b	847.2 a	653.2 b	862.3 a	672.5 b	823.1 a
Ripe fruit: sugar retention time	Long	Medium	Long	Medium	Long	Medium
Ripe fruit: sugar degree assessment	Strong	Medium	Strong	Medium	Strong	Medium

Same year with the same letters was not significantly different on two materials at 5% level.

### Molecular and ploidy date

**Figure 3:**
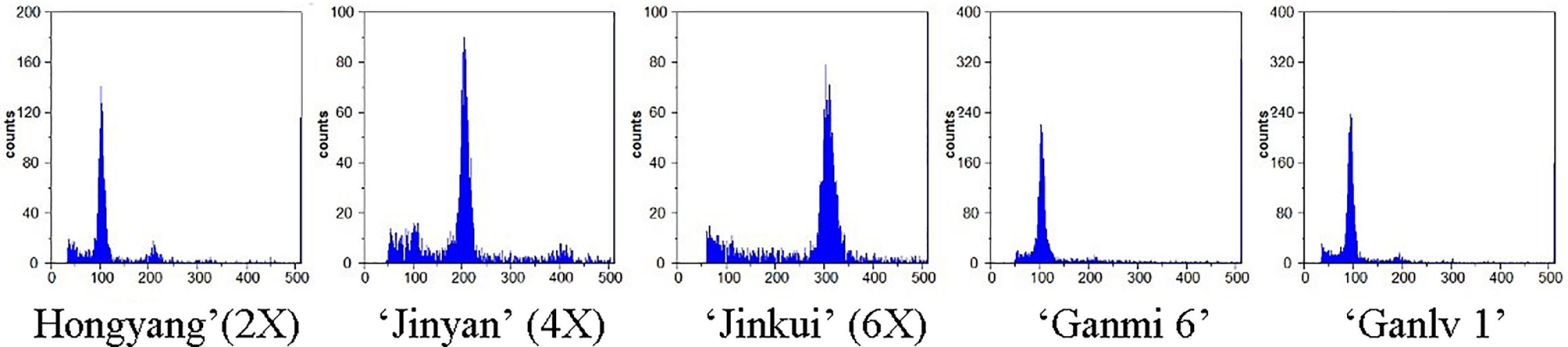
The date of ploidy test. With the known multiplier cv. ‘Hongyang’ (2 ×), cv. ‘Jinyan’ (4 ×), cv. ‘Jinkui’ (6 ×) as the standard sample, set the voltage and flow rate before test the ploidy of sample.

In order to determine whether there were differences between the two materials at the molecular level and lay a foundation for future research on QTL, SSR molecular marker technology was carried out. These two materials were completely different genotypes. The difference between the two materials according to their molecular profile as determined by presence and absence of alleles of the same molecular weight are shown in Table 3. Both ‘Ganmi 6’ and ‘Ganlv 1’ was detected as diploid, which was consistent with ‘Hort16A’ (Fig. 3).

**Table 3:** Polymorphic SSR molecular makers used to discriminate two materials.

DNA primers	Sequence	Molecular size (bp)	‘Ganlv 1’	‘Ganmi 6’
A0 55	FOR 5′TCCATTCCGCCCATCCTT3′ FEV 5′GTCCGACATTCTTGTGGTTCTG3′	150	−	−
UDK 96-019	FOR 5′ATACACTTGAAGCGCCGC3′ FEV 5′AAGCAGCCATGTCGATACG3′	100	+	+
UDK 96-039	FOR 5′GGTTTGATCGGTCTTCGAAA3′ FEV 5′ATAAATGTGTGCCAGTGCGA3′	50	−	+
UDK 96-035	FOR 5′AAGAGCCATAGCTTATTCACCG3′ FEV 5′AAGTAAAGCCATTGTCATTGCA3′	100	−	+
UDK 96-040	FOR 5′TCGAGTTACCTAGCTACTCCGC3′ FEV 5′CAAGGGAAGAAAATGTTGAACC3′	50	−	+
Est Ad-42	FOR 5′GTTAATTTGATCGGGATGG3′ FEV 5′GAGGAGCTTGAGCTGCTAT3′	150	+	−

### Dynamic changes of TS and main sugar compositions during fruit growth and development

**Figure 4:**
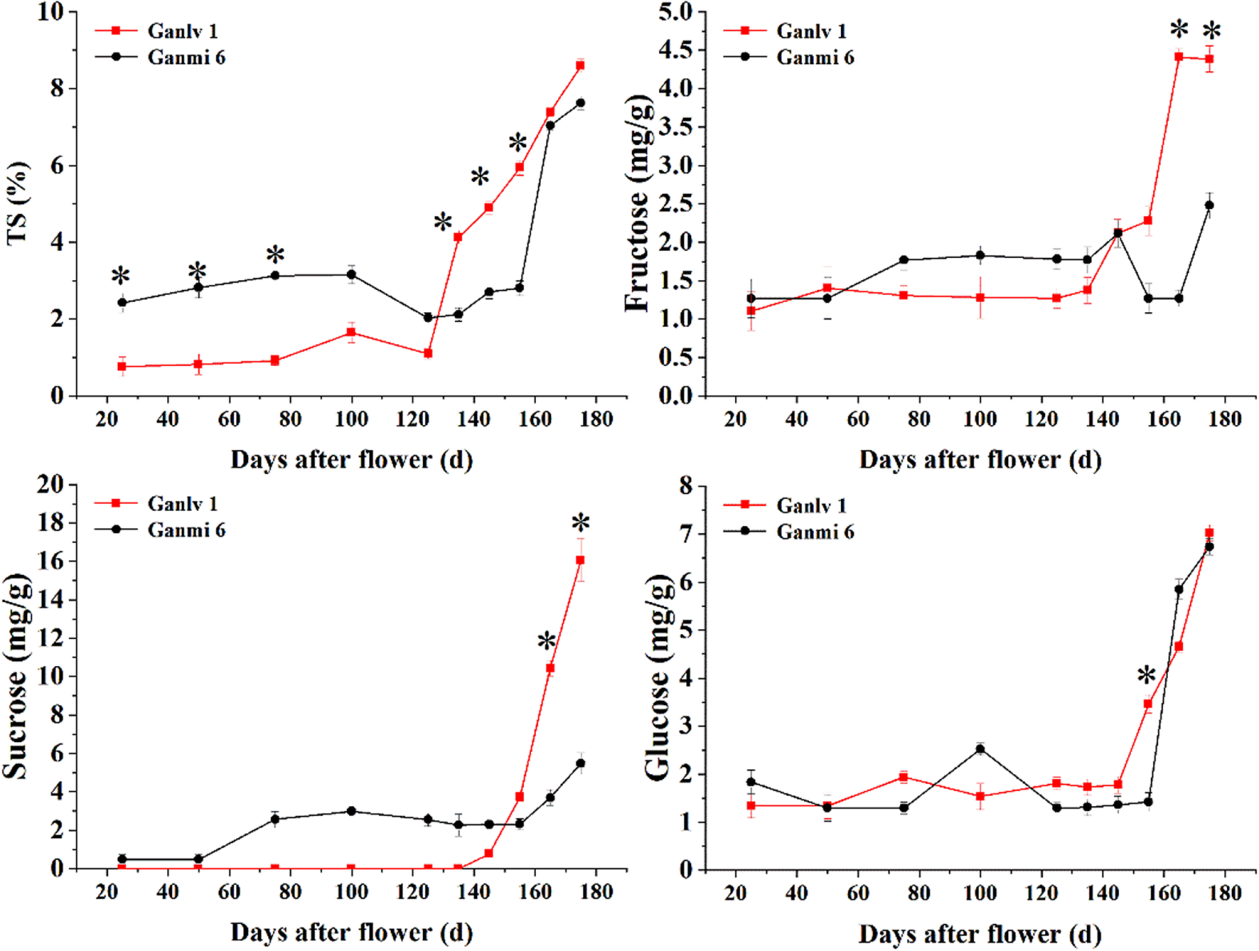
Changes of TS and main sugar compositions during its development.

We found interesting results on change of TS and main sugar compositions content during fruit growth and development. The TS of ‘Ganmi 6’ was significantly higher than ‘Ganlv 1’ before DAF125. However, the sugar composition analysis showed that fructose and sucrose in ‘Ganlv 1’ were significantly higher than that in ‘Ganmi 6’ only at DAF 165 and DAF 175, and no sucrose was found before DAF 135 in ‘Ganlv 1’. There was the same change trend of glucose content between two materials (Fig. 4).

### Gene expression of sucrose-related genes in different growth stages

**Figure 5:**
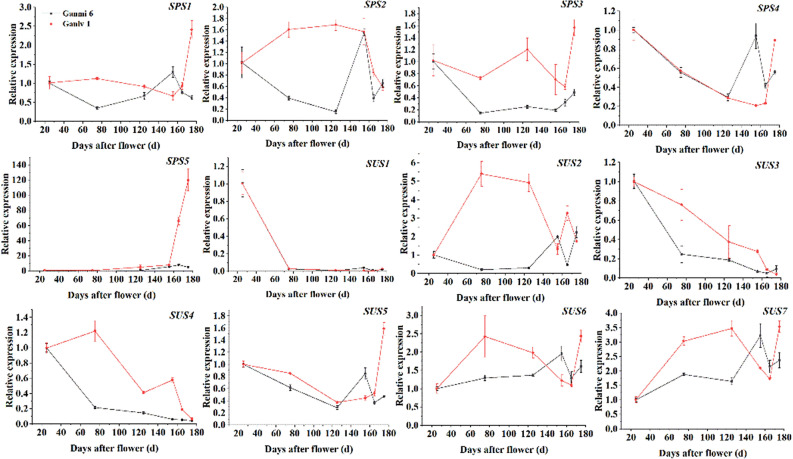
Relative expression of *AcSP*S and *AcSUS* gene family in kiwifruit.

The qRT-PCR results showed that the relative expression levels of *AcSPS1*, *AcSPS3*, *AcSPS5* and *AcSUS5* genes were consistent with the sucrose accumulation trend (Fig. 5), which was probably the main genes for the difference in sugar accumulate in different growth stages.

## Discussion

The most of *A. eriantha* are low sugar degree, but we found a high sugar degree kiwifuit from wild *A. eriantha* and observation and evaluation were carried. We found that the high sugar degree line was stable and there were uncontested differences between two materials. Sugar is an important flavor quality index of fruit, different genotypes fruit have different sugar degree, such as the ‘Hayward’ is a low sugar degree^18^ and the ‘Hongyang’ is a high sugar degree^19^. The fruit with high sugar degree are very popular in Asia, therefore, there were many kiwifruit cultivars with high sugar degree were bred by Chinese breeders in China. The weak sugar degree of fruit is the main factor restricting the promotion of *A. eriantha*. At present, the cultivated *A. eriantha* cultivars are all weak or medium sugar degree type, such as ‘White’ and ‘Ganmi 6’, their SSC were all less than 16.5%^7,20^. According to the investigation of wild *A. eriantha* in Jiangxi province of China, we found that sugar degree of most wild *A. eriantha* were low, and their fruit’ soluble solids content was lower than edible standard (14%), high sugar degree is an important breeding target for *A. eriantha*.

It is absolutely necessary to conduct DUS test for new cultivars^21^. According to the DUS test results, we found that the character of high sugar degree was a stable character. It is noteworthy that there was difference on SSC, DM and TS between the two materials on ripen fruit, these indicators were much higher on ‘Ganlv 1’ than on ‘Ganmi 6’. A large number of studies have shown that there was a significant positive correlation between DM, SSC and TS^8^. In this study, time-intensity (Osme) method was used for sugar degree assessment, the results showed that the sugar degree of ‘Ganlv 1’ was strong and retention time was long. This further proves that ‘Ganlv 1’ was a high-sugar type *A. eriantha* cultivar. The cultivars with similar genetic background have similar biological characters according to our pervious study^8^, the results of this study were consistent with pervious study, the test materials both selected from wild *A. eriantha* on Nancheng county and they have similar genetic background and biological characters. The differences between the two materials at the molecular level was determined, this can lay a foundation for future research on QTL, the genetic locus of the sugar gene will be found more accurately^22^.

The accumulation process of sugar was a very complicated process, and there were many different kinds of sugar, the most important were fructose, glucose and sucrose^23^. Fructose and glucose were monosaccharides, only sucrose was disaccharide, fruit with more sucrose was sweeter. In this study, although sucrose appeared at the later stage in ‘Ganlv 1’, the sucrose content was significantly higher than that on ‘Ganmi 6’. Rich accumulation of sucrose at the later stage was probably the main reason for the difference in sugar degree. The mechanism of large accumulation of sucrose in the later stage needs to be further studied. Studies on watermelon have shown that plants with strong light cooperation can provide more assimilates for fruit development and accumulate more sucrose^24^. There are few studies on the mechanism of high sugar in kiwifruit, and none in *A. eriantha*. Based on previous studies, we identified members of SPS and SUS gene family in *A. eriantha*. In this study, there were four genes were consistent with the sucrose accumulation trend, it is worth noting that the relative expression level of *AcSPS5* gene is extremely high in the later stage of fruit development of ‘Ganlv 1’, which is 8.25 times higher than the relative expression level of ‘Ganmi 6’. Therefore, we can infer that *AcSPS5* is a critical candidate gene. After that, the activity of enzymes related to sugar metabolism and the candidate gene will be clone^25,26^. Finally, the transgenic method will be used to determine gene function.

## Data Availability

The datasets supporting the conclusions of this article are included in the article.

## References

[1] Huang HW (2013). Chinese Kiwifruit Germplasm Resources.

[2] Zhu DY (1995). Research status and prospect of kiwifruit genetic breeding. J. Henan Agric. Univ..

[3] Xu XB, Deng YH (2000). Development and prospect of kiwifruit breeding in China. J. Jiangxi Agric. Univ..

[4] Cui ZX, Yao HY, Li Y, Huang CG (1987). *Actinidia eriantha*. Fruit Sci..

[5] Zhang HQ, Xie M, Xiao JP, Zhou LQ, Song GH (2015). Study on fruit development characteristics of *Actinidia eriantha* ‘White’. J. Fruit Sci..

[6] Zhong M (2017). The ornamental and pollinating type of *Actindia eriantha* ‘MG-15’. China Fruit.

[7] Xu XB (2015). A new easy peeling *Actinidia eriantha* cultivar ‘Ganmi 6’. Acta Hortic. Sin..

[8] Liao GL (2019). Genetic diversity of inner quality and SSR association analysis of wild kiwifruit (*Actinidia eriantha*). Sci. Hortic..

[9] Xia LX, Niu YB, Li AP (2006). Requirements and standards for phenological observation of woody plants. Shanxi Meteorol. Q..

[10] Liao GL (2019). A novel mid-maturing cultivar with high dry matter content from seedlings of ‘Jinfeng’ kiwifruit (*Actinidia chinensis*). Eur. J. Hortic. Sci..

[11] Gao JF (2006). Experimental Instruction in Plant Physiology.

[12] Wu YY (2015). Differential gene expression analysis of early-ripening mutants of grape (*Vitis vinifera* L.). Sci. Hortic..

[13] Niu J (2006). Sugar and acid contents in peach and nectarine derived from different countries and species. Acta Hortic. Sin..

[14] Wu JH (2012). Induced polyploidy dramatically increases the size and alters the shape of fruit in *Actinidia chinensis*. Ann. Bot..

[15] Wu, H. Cloning and quantitative expression of ascorbic acid synthase related genes in *Actinidia eriantha*. Dissertation, Jiangxi Agricultural University, Master thesis (2015). https://cdmd.cnki.com.cn/Article/CDMD-10410-1015648273.htm.

[16] Ampomah DC (2009). The kiwifruit lycopene beta-cyclase plays a significant role in carotenoid accumulation in fruit. J. Exp. Bot..

[17] Vandesompele J (2002). Accurate normalization of real-time quantitative RT-PCR data by geometric averaging of multiple internal control genes. Genome Biol..

[18] Mavromatis AG (2010). Molecular fingerprinting of a new kiwifruit cultivar (cv. Tsehelidis) and comparative analysis with cv. Hayward according to physicochemical properties. Sci. Hortic..

[19] Yi, C. B. A preliminary study on the biological characteristics and fruit growth and development of ‘Hongyang’ kiwifruit. Sichuan Agricultural University, Master thesis (2008). https://cdmd.cnki.com.cn/Article/CDMD-10626-2008197930.htm.

[20] Xie, M. *et al*. A new kiwifruit variety ‘White’. *Acta Hortic. Sin*. **10**, 1555+1561. 10.16420/j.issn.0513-353x.2008.10.030 (2008).

[21] Das A, Kumar D (2014). Genetic evaluation and characterization of jute (Corchorus spp. L) genotypes using DUS. Fuel Process. Technol..

[22] Ritter KB (2008). Identification of QTL for sugar-related traits in a sweet × grain sorghum (*Sorghum bicolor* L. Moench) recombinant inbred population. Mol. Breed..

[23] Mikio S (1993). Three descriptors for sugars to evaluate grape germplasm. Euphytica.

[24] Tang, Y. Study on the formation mechanism of high sugar and acid quality of ‘flavor melon’ series fruit. Huazhong Agricultural University, PhD thesis (2010). https://cdmd.cnki.com.cn/article/cdmd-10504-2010271738.htm.

[25] Liu HY, Zhu ZJ, Qian QQ, Ge ZP (2004). The effects of different rootstocks on the sugar metabolism and related enzyme activities in small and early-maturing watermelon during fruit development. Acta Hortic. Sin..

[26] Jiang N (2014). Activities of enzymes directly related with sucrose and citric acid metabolism in citrus fruit in response to soil plastic film mulch. Sci. Hortic..

